# Computational Techniques in Numerical Simulations of Arc and Laser Welding Processes

**DOI:** 10.3390/ma13030608

**Published:** 2020-01-29

**Authors:** Tomasz Kik

**Affiliations:** Department of Welding Engineering, Silesian University of Technology, Konarskiego 18A, 44-100 Gliwice, Poland; tomasz.kik@polsl.pl; Tel.: +48-32-1681

**Keywords:** FEM, numerical analyses, simulations, computational techniques, SYSWELD, displacements, stresses

## Abstract

The article presents a comparison of modern computational techniques used in numerical analyses of welding processes. The principles of the “transient” technique calculations with a moving heat source, the “macro-bead” (MBD) technique, with an imposed thermal cycle on a selected weld bead section and the “local–global” approach with shrinkage calculation technique were described. They can be used, depending on the variant chosen, both for individual, simple weld joints and those made of many beads or constructions containing dozens of welds and welded elements. Differences in the obtained results and time needed to perform calculations with four different calculation examples of single and multipass arc and laser beam welding processes were presented. The results of calculations of displacements and stresses distributions in the welded joints using various computational techniques were compared, as well as the calculation times with the described techniques. The numerical analyses in the SYSWELD software package have shown the differences between the described computational techniques, as well as an understanding of the benefits and disadvantages of using each of them. This knowledge allows preparing an efficient and fast optimization of the welding processes, often aimed at minimizing deformations in the first place, as well as detection of potential defects of both simple and complex welded structures. In general, the possibilities and flexibility of modern numerical calculation software have been presented.

## 1. Introduction

Terms such as simulation, calculations or numerical solutions have been known to engineers for a long time, not only in technical fields but also in medicine or natural sciences. Of course, with the development of electronics, at this time, great progress was made both in terms of the hardware itself and the possibilities that the modern software offers us. Many branches of technology can now show almost comprehensive numerical solutions, from the design stage, production preparation, through the stage of determining technological and production conditions, to the overall optimization of the production process. It is also possible to simulate the actual or assumed conditions of use of the product together with determining its durability. The use of modern manufacturing techniques such as laser or electron beam welding places requirements that are increasingly difficult to meet. Increasing requirements regarding the accuracy of element preparation and maintaining minimum dimensional deviations during and after the welding process mean that we readily use modern computational tools [1,2,3,4,5,6,7].

The development of numerical analysis software has recently focused mainly on advanced second-generation simulation calculations. These types of simulations support a unique way of creating the final product using various industrial technologies. In this way, it is possible to simulate the entire production process and its relationships with the limit states of structures and materials. This is a typical path, e.g., in the automotive industry, where the processes of sheet metal forming, body welding and in the last stage of crash tests are analyzed. However, in the case of numerical analyses of welding processes, despite the long and intensive development of computational techniques, it is still difficult to achieve all of the mentioned operations in one calculation program [1,2,3,4,5,8].

Since welding is one of the most widespread technological processes, this technology has also received a lot of attention when developing modern simulation tools. The problem with the simulation of this process is the fact that the most frequently moving heat source is the reason for the uneven distribution of displacements and stresses in welded elements [5,6,7]. However, they are not only dependent on the moving heat source itself. Their values and distributions are also strongly dependent on many other technological factors such as clamping condition, mechanical and thermal properties, type of the welding technology and welding parameters, preheating temperatures, weld joints design, temperature of surroundings, etc. The residual stresses in the structure after welding have a negative influence on the durability of the structure and its reliability under various operating conditions. The degree of complexity of the process itself and the non-linearity of phenomena that occur in the welded element during its heating and cooling, and their dependence on the type of material and technology used, significantly increase the degree of complexity of numerical analyses [5,6,7,9,10].

Currently, there are two views on the issue of conducting and solving tasks in the field of numerical analysis of welding processes. The first of them is associated with a detailed, often partial solution of selected effects of the welding process, consisting of determining the shape and dimensions of the weld molten pool, heat affected zone (HAZ) and related metallurgical and mechanical changes in the area of the welded joint [1,2,3,4,5]. The result of this type of analysis is always a detailed description of the solution, unfortunately only in relation to a specific, individually considered data and process parameters. The second approach is to apply numerical analyses to large, complete parts and production units. Due to the complexity of the structure, this method must contain a number of simplifications, which to some extent reduce the resulting accuracy of calculations. Despite this disadvantage, this allows conducting analyses that in the first approach, even despite the currently available computing power, are practically impossible to perform, precisely because of the number of variables that should be taken into account. Not without significance, in this case, is the constant focus on reducing production costs and reducing to the absolute minimum the time needed to prepare a new product [11,12,13,14]. 

The use of numerical analyses today allows for a better understanding of the production process, finding relationships that connect individual process parameters to its results and increases product quality. It also leads to a significant cost reduction in the production preparation stage by limiting and often excluding costly prototypes. This is particularly important when making samples or prototypes involving a lot of material, time and are energy-consuming. Based on the results of numerical simulations, it is possible to determine the optimal solution, even if their results are not 100% consistent (in terms of value) with the results of real tests. Thanks to the constant development of modern simulation software, it offers us today various possibilities of approach to the issue of numerical analyses, allowing for the mentioned analyses within both one joint and entire constructions. In the following, based on the SYSWELD software from ESI Group, typical computational techniques are presented. Each of them are successfully used in today’s modern computational packages [1,2,3,4,5,8,11,12,13,14,15,16,17,18].

## 2. Description of the Problem

As it was already mentioned, depending on the selected calculation method, numerical analyses of welding processes carried out in the SYSWELD environment can be divided into the following groups:

Local analysis: Used to determine the distribution of temperature fields, metallurgical phases as well as stresses and deformations within one welded joint;

Global analysis: This applies to the entire structures and usually involves changing the size of the structures (displacements) and stresses distributions around the welds [1,2,3,4].

In the case of welding, essentially the only load on the workpiece is the non-stationary distribution of the temperature field both on its surface and in many cases in thickness. Thus, the temperature in the model is determined by means of a variable function in time and dimensional space. Accurate determination of the temperature field distribution is, therefore, the basic and most important step in determining its influence on the metallurgical properties of the material and the stresses and deformation distributions of the welded structure. In order to refine the record of changes in temperature function in time and space, it is necessary to define a mathematical model of a heat source [1]. This description, however, brings with it a lot of problems due to the complexity of the issue. There is a need to take into consideration many input parameters and apply some simplifications in the description of this model. When creating it, the influence of convection in the liquid metal pool, active elements, type of protective gas, wire diameter or method of transferring metal in the arc are not taken into account. Models of heat sources can be divided into three basic groups while defining the degree of complexity of the analyses carried out [1]:

One-dimensional;

Two-dimensional;

Three-dimensional.

Therefore, depending on the dimension of the heat source model used, point, linear or flat models of heat sources were used in the analyses. Their advantage was low “resource consumption” in the calculations. The limitations of these heat source models resulted primarily from the available computing power. Nowadays, when we have sufficient computing power, in most cases we almost exclusively use three-dimensional models [5,11,12,13,14,15,16,17,18]. 

### 2.1. Transient Technique: Moving Heat Source

**Figure 1:**
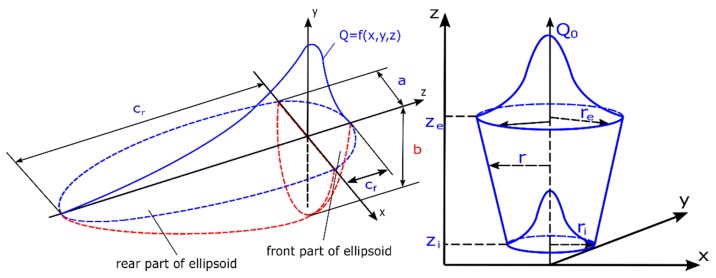
Double ellipsoid and 3D conical heat source models [1,5,19].

In the case of local analyses, when the moving heat source model is used, the calculations are carried out by the so-called “transient” technique, also called the “step-by-step” technique. Calculations are made in this case for each subsequent time increased by a given time step, which is automatically adjusted depending on the mesh density of the model or set by the user. The mathematical model of the heat source in this type of analysis moves along the line that determines the welding trajectory. The parameters used to describe the heat source model, in this case, are the actual welding process parameters, i.e., welding current, arc voltage, welding speed or thermal efficiency of the method, etc. So, this is mostly data that can be found, among others, at the Welding Procedure Specification Form (WPS) but also are part of the knowledge of every welding engineer. One of the most popular models used in current numerical analyses is the volumetric double ellipsoid model, also known as Goldak’s model [1,5,15,16,17,18,19,20]. It consists of two ellipsoids which, placed in two perpendicular planes, create an image of the weld pool in both horizontal and vertical space (Figure 1). Transferred heat into a volume is described by Equations (1) and (2) [1,5,19,21]: 

For the front part of the heat source model, the Equation is:

(1) $$Q_{f}\left(x,y,z\right)=\frac{6\sqrt{3}f_{f}Q}{abC_{f}\pi \sqrt{\pi }}exp\left(\frac{-kx^{2}}{a^{2}}\right)exp\left(\frac{-ly^{2}}{b^{2}}\right)exp\left(\frac{-mz^{2}}{c^{2}}\right)$$

and for the rear part of the heat source model, the Equation is:

(2) $$Q_{r}\left(x,y,z\right)=\frac{6\sqrt{3}f_{r}Q}{abC_{r}\pi \sqrt{\pi }}exp\left(\frac{-kx^{2}}{a^{2}}\right)exp\left(\frac{-ly^{2}}{b^{2}}\right)exp\left(\frac{-mz^{2}}{c^{2}}\right)$$

where *Q_f_*, *Q_r_* are volumetric heat flux density in front and rear part of the model [W/m^3^]; *Q* is total power source; *a*, *b*, *c_f_*, *c_r_* are width, depth and length of the front and the rear part of the molten pool; *f_f_*, *f_r_* are constants which influence energy flow intensity into the material (*f_f_* + *f_r_* = 2); and k, l, m are coefficients enabling modification of the liquid metal pool shape.

The efficiency of the heat transfer into parent material is given by the applied welding method [20]. For the proper heat source calibration, it is necessary to compare the results with experimentally determined and measured values. It is mainly the overall heat, the geometrical parameters of the molten pool and the efficiency of the heat transfer from the source into the material. After specifying the input parameters, it remains to add the constants *f_f_*, *f_r_* affecting the distribution of energy flow to the material and choose the coefficients k, l, m. Practice shows that we usually accept intercourse of *f_f_* to *f_r_* as 60:40. The choice of coefficients k, l, m is more complicated. If the unmodified heat source model is used, all three factors will be equal to three. The unmodified source is particularly suitable for simulated welding with coated electrodes. In the case of welding simulation, e.g., with the GMAW method, the model must be modified to obtain the correct shape of the liquid metal pool and the values of the coefficients chosen experimentally [1,5,15,16,17,18,19,20,21,22].

While Goldak’s source successfully covers the scope of numerical analyses of arc welding processes, in the case of laser or electron beam welding, the conical model with a Gaussian distribution is currently most commonly used. Its conical shape allows good reproduction of welding processes carried out using welding heat sources with a high concentration of energy (for example laser and electron beam welding). The mathematical description of this model can be represented by two Equations [1,5,22]:

(3) $$Q\left(x,y,z\right)=Q_{0}exp\left(-\frac{x^{2}+y^{2}}{r_{0}^{2}\left(z\right)}\right)$$

(4) $$r_{0}\left(z\right)=r_{e}+\frac{r_{i}-r_{e}}{z_{i}-z_{e}}\left(z-z_{e}\right)$$

where *Q*_0_ is the maximum value of volumetric heat flux density; *r_e_*, *r_i_* are upper and lower 3D cone radius dimensions parameters; *z_e_*, *z_i_* are the 3D cone length parameters; and *x*, *y*, *z* are point coordinates (Figure 1).

Equation (3) describes the volumetric heat flow density into the material in the dependence of coordinate data. Equation (4) supplements Equation (3) by the definition of the radius change in the direction of the depth. 

**Figure 2:**
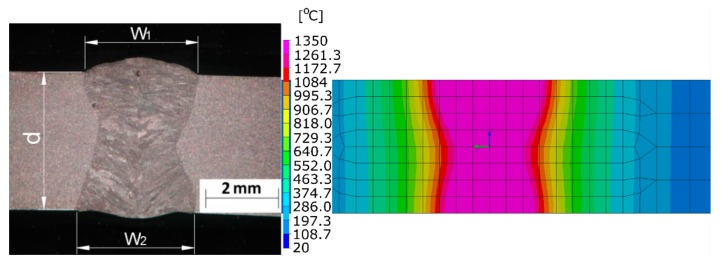
An example of heat source model parameters calibration. Comparison of the calculated shape and size of the molten zone in the laser welding process with a real macrograph of laser weld bead (laser beam power 2000 W, welding speed 0.5 m/min).

This way of defining a heat source model and conducting numerical analyses based on a moving heat source requires its appropriate calibration procedure (Figure 2). Only in this case will it be possible to recreate with it the actual distribution of temperature fields that affect the metallurgical transformations, stress and deformation distributions that occur. It is a long-term process but can be carried out on partially simplified models (including 2D cross-section models). Incorrect calibration of the heat source model leads to duplication of errors during this stage and simulation, which in turn leads to incorrect results.

The analysis carried out using the “transient” technique is divided into three parts: preparation of the material base, calculation of thermal phenomena and related metallurgical changes as well as mechanical phenomena (i.e., stresses and displacement distributions, etc.) [1,2,3,4]. Taking into account the fact of performing calculations at each subsequent time, the user after completing the analysis receives a powerful set of thermo-metallurgical and mechanical data related to the simulated process. The price of such a complex solution is the extended duration of calculations. This is the main reason why the calculation of large welded structures with many welds uses other calculation techniques. However, such a large amount of data, including distribution of temperature fields, hardness, metallurgical phases, stresses and deformations makes it an ideal method by which the already mentioned local effects of the welding process can be determined. Based on the results of these analyses, it is also possible to conduct further simulations regarding the impact of external loads, i.e., forces, moments and pressures, as well as e.g., assessment of fatigue life of a structure based on the Dang Van criterion [1,2,3,4,16,18].

### 2.2. Macro-Bead Technique: Imposed Thermal Cycle

**Figure 3:**
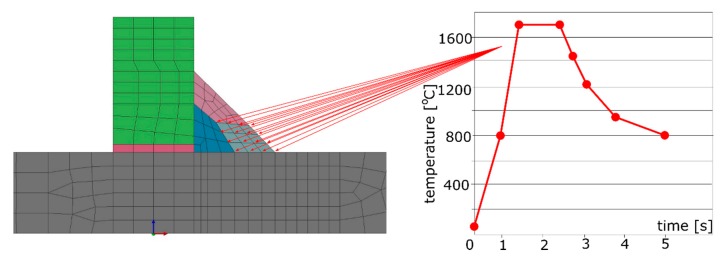
Imposed thermal cycle set on selected nodes of the model in calculations using the macro-bead (MBD) technique.

As it was already mentioned, calculations using the “transient” technique are practically impossible to use, from the point of view of the amount of data generated during them, in the case of numerical analyses of large or complex welded structures. In such cases, the “macro-bead” (MBD) technique is used [1,3,4,23,24], which is in a way an extension of the “transient” technique and involves the use of a properly prepared thermal cycle immediately on one or several areas (elements) of the model simultaneously (Figure 3). 

In this technique, the actual welding trajectory is divided into sub-areas so that the welding order and directions are maintained. The number of these sub-areas and the time step are defined on the basis of technological parameters of the welding process as well as the experience of the person working with this method [1,2,3,4]. The standard procedure is to prepare the thermal cycle based on a simple model “transient” analysis result (often using also 2D cross-section models) or as a result of thermocouple measurements. This cycle is then applied successively to the mentioned sub-areas of welds.

**Figure 4:**
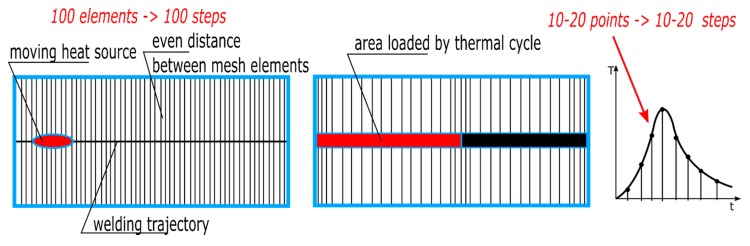
Differences between the number of simulation time steps for the “transient” and MBD analysis.

The difference is that in the case of the “transient” technique with a moving heat source, it is needed as many time steps (calculation cards) as there are elements in the grid on the welding trajectory. When applying the imposed thermal cycle, required are as many steps as the points describing the given thermal cycle in each of the sub-areas (Figure 4). In addition, the mesh can be modified and divided into a significantly smaller number of finite elements because there are no temperature gradients in the middle of the weld section at the time of the applied thermal cycle. They only occur at the beginning and end of the bead (sub-section). Therefore, in the MBD technique, the mesh is only refined at the edges of the section of elements on which the thermal cycle is applied. In their center, due to the lack of thermal gradient, the mesh is much thinner. Thus, for example, 100-time steps in the calculation with the “transient” technique can be replaced with a maximum of 20–40 time steps in the MBD technique, which significantly reduces the calculation time (Figure 4).

Based on the MBD technique, it is also possible to perform calculations using the “local–global” method [1,3,4]. Its main idea is the assumption that the welding process leads to local changes in stresses distribution and plastic deformations, while the effect of this on a global scale is a specific state of deformations. The local effects of the welding process are determined in this case by means of precise calculation models of single welded joints using the “transient” or MBD techniques. When the joints are repetitive (welded with the same parameters), the calculations are significantly accelerated. The results of local analyses are then transferred to the global model to determine the total deformations of the structure. The limitation of this method, however, is that the results of the analyses carried out on the global model are only structural deformations as well as internal forces and moments under specific mounting conditions. The stresses level and distribution of individual metallurgical phases are determined by local models obtained as a result of analyses carried out using the “transient” or MBD technique [1,3,4,23,24].

### 2.3. Shrinkage Technique: Distortion Engineering

**Figure 5:**
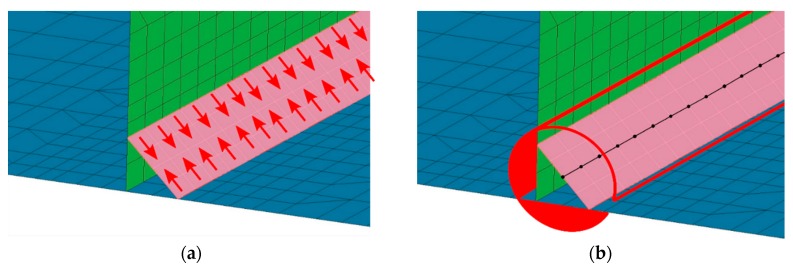
View of a fragment of the T-joint model with (**a**) a representative shrinkage force and (**b**) marked trajectory (weld axis) with shrinkage area (tube) [4].

The application of the “local–global” method in the case of very large and complicated welded constructions may have been problematic even despite the acceleration of calculations, which is provided by the use of the MBD technique [1,3,4]. In this case, it is also possible to use the “shrinkage” technique, which, despite many simplifications, allows to determine the deformation of the structure in situations where the standard techniques and even MBD is not possible to carry out due to too long calculations time. In accordance with the assumptions of the “local–global” method, in the case of calculations carried out using the “shrinkage” technique, calculations of thermal phenomena and related metallurgical changes are not carried out. Only the results of the analysis of mechanical phenomena occurring during the welding process are used. In a simplified way, it can be described that the thermal cycle first causes an expansion of a certain area of the material as a result of its heating and then shrinkage, which is caused by specific cooling conditions and heat dissipation from this zone. If during the cooling process some areas of the joint are subject to shrinkage, it is the user’s task to find a representative value of the force that causes this shrinkage. In order to find this representative force causing the contraction, it is necessary to carry out the calibration process. For this reason, a special local model is created (for individual, selected joints from the whole structure), on which a classic “transient” analysis is performed and deformations are determined in specific places of the model. Then, the same model is calculated using the “shrinkage” technique. The deformations obtained as a result of the analysis are compared to the model from the classical analysis and the input data are calibrated to obtain similar values. The parameter that changes the value of the representative force for this calculation method is the radius of the pipe/sphere encompassing the model grid nodes at a certain distance from the assumed trajectory of the weld (weld axis) (Figure 5). All mesh nodes inside the area enclosed by its radius will be subjected to contraction. By increasing the value of this radius, it is possible to set the conditions of the shrinkage impact in such a way that the results of the local model of this technique come close to the results obtained in the local analysis of the “transient” type. After the calibration, it can be transferred to the model of the entire structure. In the case where the structure has a large number of the same joints (joint type and lateral dimensions of the weld), due to its repeatability, the calculation process is significantly accelerated in time as it was the case with the “local–global” method using MBD techniques [1,2,3,4,23,24].

Analyses carried out using the “shrinkage” technique allows for quick analysis of structure deformations with a very large number of welded and welded joints, e.g., with a laser beam, as well as optimization of the order of their execution in terms of minimizing deformations. The method allows for an efficient search for a compromise between the clamping conditions (the degree of stiffening of the structure) and the level of stresses and deformations as well as determining the optimal welding sequence, which can additionally be a complicated, complex sequence of variable fastening of elements and increasing rigidity of the structure as the assembly progresses [1,3,4,23,24].

## 3. Calculation Examples

To show the differences between the results and the duration of calculations obtained using individual techniques, as well as to introduce the described issues, several examples of analyses were carried out using the techniques described above. All calculations were made on a computer with an Intel i5-8750H processor and 16 GB of operational memory in VisualWeld 14.5 (SYSWELD 2019.0 solver produced by ESI Group, Paris, France). 

### 3.1. Numerical Analysis of a T-Joint with One Fillet Weld Arc Welding Process

**Figure 6:**
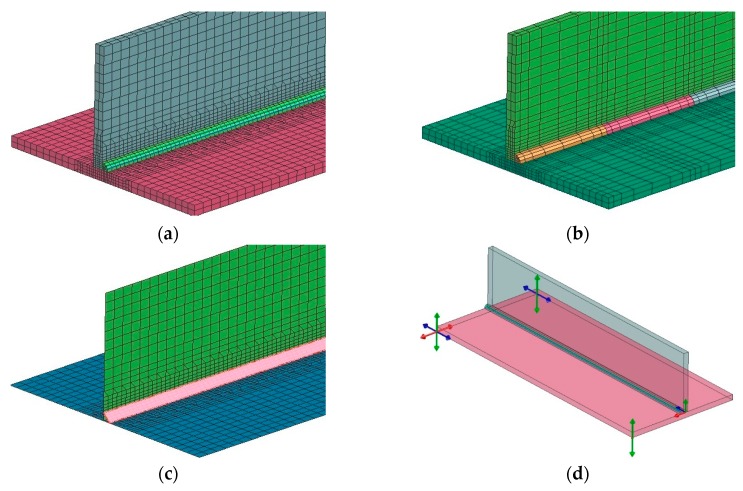
Finite element (FE) models prepared for the comparison of computational techniques for (**a**) “transient”, (**b**) MBD, (**c**) “shrinkage” techniques and (**d**) clamping conditions of welded elements.

**Figure 7:**
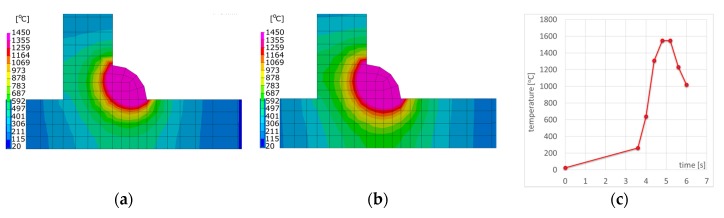
Temperature fields distributions comparison on the cross-sections of arc welded T-joint: (**a**) calculated using the “transient”, (**b**) MBD technique and (**c**) an example of imposed thermal cycle used in the MBD technique calculations.

In order to compare the results obtained with the described techniques, a calculation model of the T-joint of sheets 4.0 mm thick and 200 mm long made of S355 steel GMAW welded with one fillet weld was prepared. As a material database was used standard S355 database included in the SYSWELD material database. The finite element (FE) model consisted of 26,172 elements and 20,923 nodes in the case of calculations using the “transient” method, and the heat source model used was a double ellipsoid model. Heat source parameters were set as follows: energy per unit length, 380 J/mm; welding speed, 5.0 mm/s; arc heat efficiency, 0.7; length/width/depth of Goldak’s model, 8.0/4.0/2.0 mm. In the case of calculations with the imposed thermal cycle, the FE model consisted of 13,982 elements and 11,193 nodes, and in the case of calculations using the “shrinkage” method, respectively, 4550 2D elements and 4464 nodes (Figure 6a–c). The thermal cycle used in the calculation of the MBD technique and the calibration of the shrinkage method model was carried out for auxiliary analysis using the 2D cross-section model (Figure 7). Mesh was fined in the weld area to increase calculation accuracy. Clamping conditions during welding were set to simulate welding without any additional mounting. It means that were used three nodes in which the possibilities of displacement in three, two and one of the directions were blocked (Figure 6d). This type of boundary condition simulates a situation where the elements lie freely on the welding table without additional clamping during the welding and cooling process. Boundary conditions correspond to heat dissipation to the environment on each external surface and the heat transfer coefficient correspond to the cooling conditions in the free air with an ambient temperature of 20 °C.

**Figure 8:**
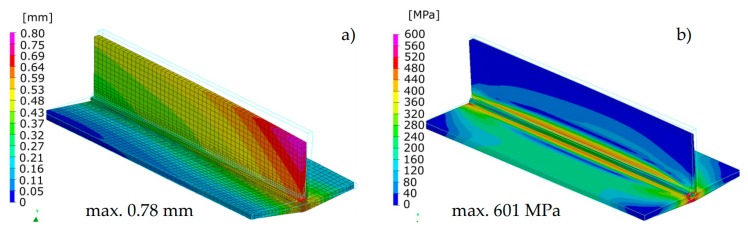
Distributions of (**a**) normal displacements and (**b**) von Mises stresses in arc welded T-joint calculated using the “transient” technique.

**Figure 9:**
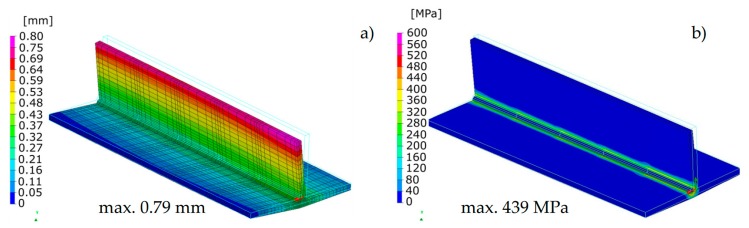
Distributions of (**a**) normal displacements and (**b**) von Mises stresses in arc welded T-joint calculated using the Macro-Bead Distortion Engineering (MBD DE) technique.

**Figure 10:**
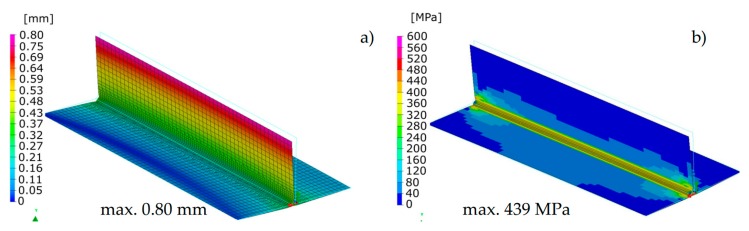
Distributions of (**a**) normal displacements and (**b**) von Mises stresses in arc welded T-joint calculated using the “shrinkage” technique.

The calculated distortion distributions in all three techniques differed to some extent in the size distribution, but the maximum values and the deformation tendency of the joint were similar (Figure 8, Figure 9 and Figure 10). As was observed, the stresses values in the case of the “transient” technique were higher; however, the maximum value is usually influenced in this case by the beginning and end of the weld, while in the remaining areas they are much lower (Figure 8). It also results in a sense from the definition of the start and end of the heat source model movement (certain inaccuracies related to the definition of the amount of heat in these areas can be improved by using a power ramp). This difference cannot be seen in the other two other techniques. However, when the “macro-bead” technique is used, there are characteristic peaks of the stresses values at the points where the subsequent sections are joined at which the heat cycle is given (Figure 9 and Figure 10). The precision of stresses state calculations for these last two methods is much lower due to the simplifications used and should rather be treated as approximate values.

Depending on the calculation method used, obtaining the result requires a certain time. A comparison of calculation times is presented for all variants in Table 1. If the MBD technique is used, it is also possible to carry out the “local–global” method (without the calculation of the temperature and metallurgical phases distributions), therefore the time for this case is also given as Macro-Bead Distortion Engineering (MBD DE). It should also be added that the times needed to calibrate the computational models in MBD and the “shrinkage” technique were not included. However, these operations can be carried out on simplified models, e.g., 2D cross-section (same as for the “transient” heat source model calibration), which significantly reduces the computation time compared to the variant using the classical “transient” technique. In the case for the complex structure wherein there are many of the same joints/welds, a significant acceleration of calculations is visible because the calibration on a separate model is made only for one, especially when the changes do not apply to technological parameters (welding current, welding speed, etc.).

### 3.2. Numerical Analysis of the T-Joint Multipass Arc Welding Process with Three Fillet Welds

**Figure 11:**
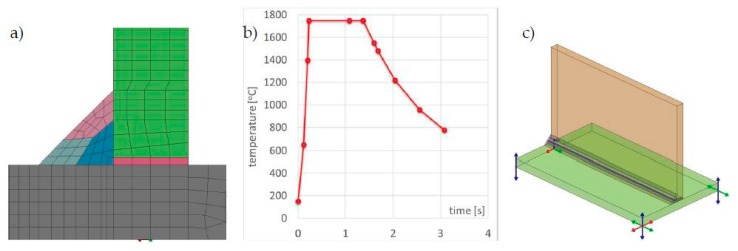
A view of (**a**) the 2D cross-section of the FE model, (**b**) imposed thermal cycle prepared for the calculation by the MBD technique and (**c**) clamping conditions of welded elements.

**Figure 12:**
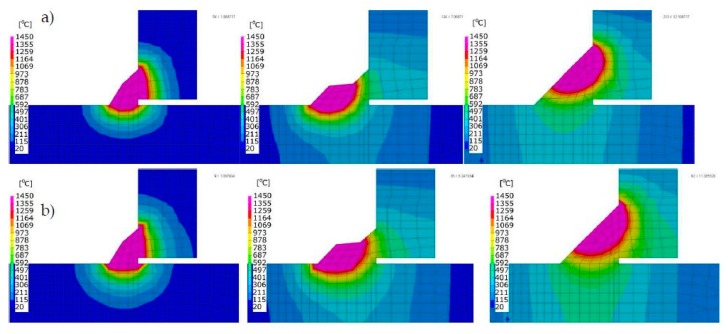
Comparison of temperature field distributions on the cross-sections of the multipass T-joint calculated using the (**a**) “transient” and (**b**) MBD techniques.

In the case of multipass welded joints, it is not possible to use the models made in the shell technique (made of 2D elements). However, it is still possible to use the MBD technique as a tool that significantly reduces computation time allowing the analyses of more complex structures. Therefore, a T-joint model was prepared for S355 steel sheets 10 mm thick and 200 mm long, GMAW welded on one side, with three fillet welds. In both computational techniques, the same model (without changing the density of the mesh inside the sections, characteristic of the MBD technique) was used as a computational model consisting of 49,780 elements and 41,814 nodes. For the material database, the standard S355 database included in the SYSWELD material database was used. Boundary conditions replies to clamping conditions during welding were set to simulate welding without any additional mounting as it was described in the previous example and shown in Figure 11. Boundary conditions corresponding to heat dissipation to the environment also were on each external surface as free air cooling and the model was cooled to ambient temperature at a level of 20 °C. The calibration of the heat source model was carried out on the 2D cross-section model and on this basis, the heat cycle for MBD calculation was also prepared (Figure 11 and Figure 12). As the heat source model in the “transient” technique was used Goldak’s heat source model. Heat source parameters were set as (similar for each bead): energy per unit length, 450/350/390 J/mm; welding speed, 8.0 mm/s; arc heat efficiency, 0.7; length/width/depth of Goldak’s model, 12.0/8.0/4.0 mm. The area of elements affected by the heat source model (in the SYSWELD nomenclature called LOAD) was additionally determined separately to get the right expected shape of the molten area of each bead.

**Figure 13:**
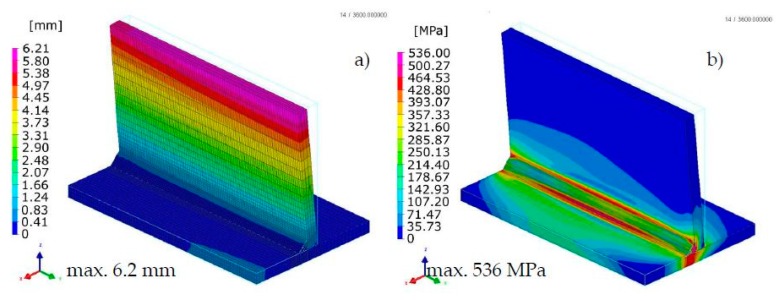
Distributions of (**a**) normal displacements and (**b**) von Mises stresses in arc welded multipass T-joint calculated using the “transient” technique.

**Figure 14:**
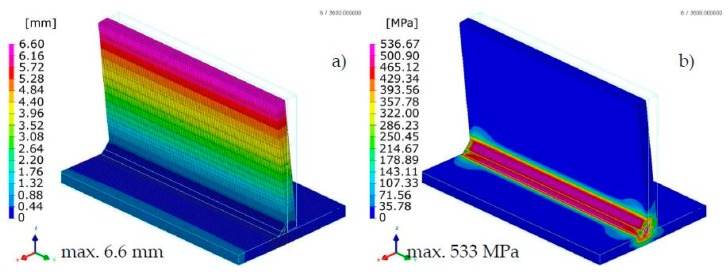
Distributions of (**a**) normal displacements and (**b**) von Mises stresses in arc welded multipass T-joint calculated using the MBD technique.

The calculated displacements, similarly to the previous case, slightly differed in the size and value distribution; however, they are similar to the place and direction of occurrence (Figure 13 and Figure 14). 

The calculated stresses values were also similar. As for the distribution of these stresses, also in this example, the “transient” technique offers more precise results, while in the case of the “macro-bead” technique, the user also receives more general information about the order of height and location of these stresses in the joint (Figure 13 and Figure 14). In addition, in the case shown, no value peaks are visible along the length of the joint because the heat cycle has been applied along the entire length of each simulated beads (Figure 14).

**Figure 15:**
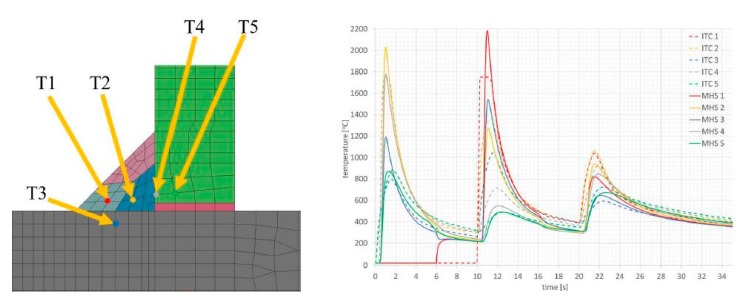
Thermal cycles in selected nodes of the model, calculated using the “transient” (moving heat source) and MBD (imposed thermal cycle (ITC)) techniques.

The summary of calculation times by the compared techniques/methods are presented in Table 2. A comparison of thermal cycles of the welding process calculated by the “transient” and MBA techniques showed considerable similarities in their course as well as values (Figure 15).

### 3.3. Numerical Analysis of the Laser Welding Process of Pipes Butt Joints

When using laser techniques, it is also possible to use the described computational techniques. Especially in this case, when the number of welds made using a laser beam is large and they are often repetitive welds, optimization of the order of the execution of individual beads is an ideal task to perform using the technique of “shrinkage” in the “local–global” approach. The use of the “transient” technique due to the need to use a very dense mesh (due to the size of the laser beam focus point) causes a drastic increase in the calculation time due to a large number of analysis time steps. However, typical issues can also be solved using the MBD technique, which efficiently reduces this time.

**Figure 16:**
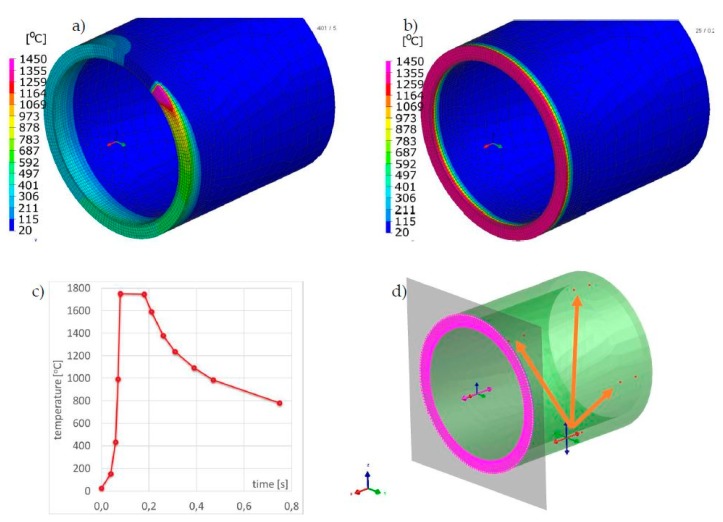
Temperature fields distributions during the laser welding process of pipes butt joints: (**a**) calculated with “transient”, (**b**) MBD technique, (**c**) imposed thermal cycle used in MBD technique calculations and (**d**) clamping conditions during welding.

For numerical simulations of 70 × 5.0 mm pipes laser butt welding, a calculation model consisting of 16,576 elements and 14,232 nodes was prepared. Simulations were done for two different materials: AISI304 stainless steel and Inconel 625 nickel superalloy, which are also implemented in the standard SYSWELD material database. The calculation model was made as a symmetrical model (only half of the joint separated by a plane of symmetry was calculated to reduce the calculation time). Calibration of the heat source model was carried out on the 2D cross-section model, and on this basis, the imposed heat cycle used further in the MBD calculation was also determined (Figure 16a–c). Boundary conditions replies to clamping conditions were set as a complete stiffening of the two nodes in every four places (quarters) of the tube as shown in Figure 16d. After cooling the element in the final phase of calculations, the element was released from its mounting. For the clamping boundary conditions, in this case, a symmetry condition was also defined at the contact surface of the axisymmetric model. Heat dissipation to the environment was set on all external surfaces of the model as the heat transfer coefficient corresponding to the cooling conditions in the free air with an ambient temperature of 20 °C. In this case, described earlier, the conical source with a Gaussian distribution was used as a moving heat source model in the “transient” calculation technique. Heat source parameters were set as follows: laser beam power–3.0 kW, welding speed–39 mm/s, laser beam heat efficiency–1.0, z_e_–5.0 mm, z_i_–0 mm, r_e_–1.0 mm, r_i_–0.4 mm. For MBD calculation was set the thermal cycle obtained as a result of heat source calibration on a simple 2D model. 

**Figure 17:**
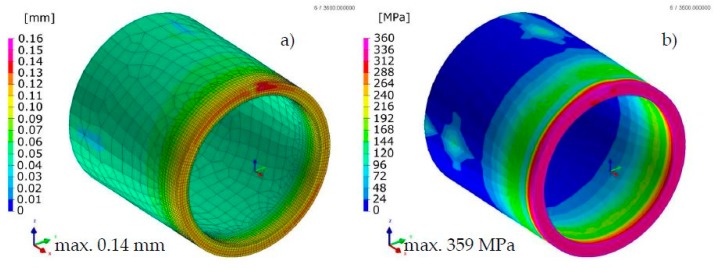
Distributions of (**a**) normal displacements and (**b**) von Mises stresses in stainless steel laser-welded pipes butt joints calculated using the “transient” technique.

**Figure 18:**
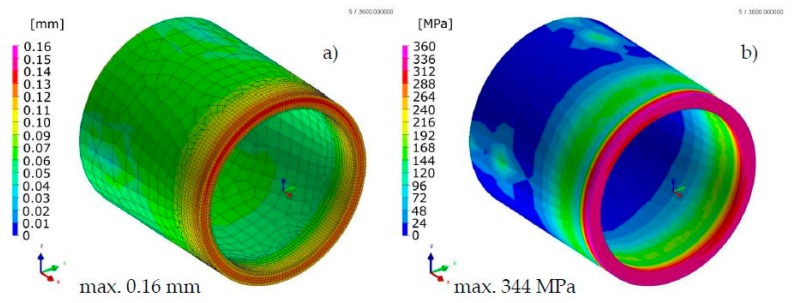
Distributions of (**a**) normal displacements and (**b**) von Mises stresses in stainless steel laser-welded pipes butt joints calculated using the MBD technique.

**Figure 19:**
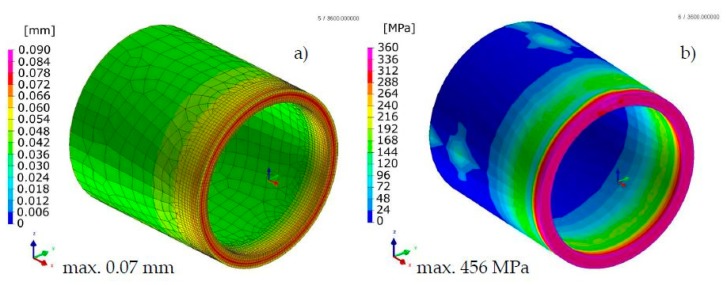
Distributions of (**a**) normal displacements and (**b**) von Mises stresses in Inconel 625 nickel superalloy laser-welded pipes butt joints calculated using the “transient” technique.

**Figure 20:**
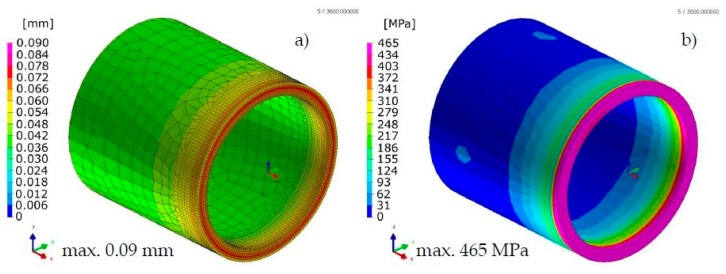
Distributions of (**a**) normal displacements and (**b**) von Mises stresses in Inconel 625 nickel superalloy laser-welded pipes butt joints calculated using the MBD technique.

**Figure 21:**
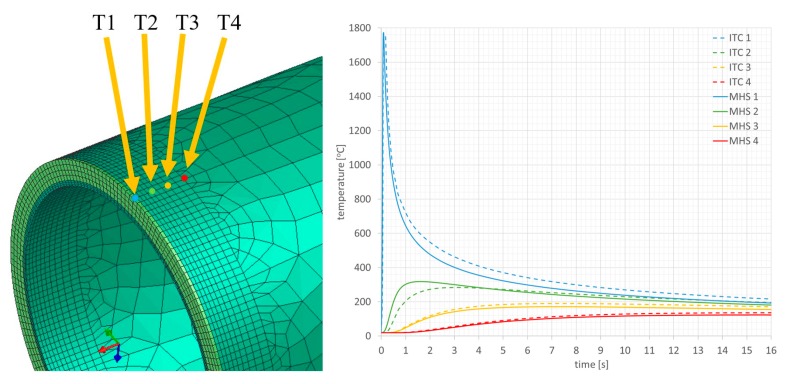
Thermal cycles in selected nodes of the model, calculated using the “transient” (moving heat source) and MBD (imposed thermal cycle (ITC)) techniques for AISI 304 pipes laser welding simulation example.

Comparison of the results calculated with both techniques showed similarities in the values and the distributions of displacements and stresses in the analyzed welded joints (Figure 17, Figure 18, Figure 19 and Figure 20). The thermal cycles of the welding process calculated by the “transient” and MBD techniques showed a high convergence of their course as well as values (Figure 21). The summary of calculation times achieved by different calculation techniques/methods are presented in Table 3.

## 4. Conclusions

This article describes three calculation techniques for welding processes numerical simulations using the finite element method. The described methods are both the “transient” method that gives the largest amount of precise information and details. Due to their specificity, they require conducting step-by-step calculations, which increases the calculation time, as well as the MDB or “shrinkage” technique, which in turn allow for significant reducing the calculation time and allows conducting analyses of complex welded constructions at the expense of quality and precision of obtained results. Significantly longer calculation times in the “transient” technique result from the aforementioned calculation method, where the moving heat source travels over the grid elements generating subsequent time moments (calculation cards) as well as the fact that all quantities describing the welding process are calculated in these steps (temperature fields, phase distributions, heat fluxes, etc.). In the case of complicated structures with a large number of welds/joints, this makes it impossible to carry out analyses due to the real-time needed for their implementation and the amount of data generated in the resulting output file. However, analyses of this type result in a number of important, precise data on the so-called local welding process effects for the selected welded joint. They also support the creation of simple (and therefore requiring shorter calculation times) models for calibrating the input data used in the MBD or “shrinkage” technique. They can be both 2D cross-section models as well as fully three-dimensional models, however, with less complexity or smaller size (primarily length but sometimes also transverse dimensions).

As the presented examples have shown, the use of the MBD technique significantly reduces the time of calculations due to the fact that in this technique the nodes of the mesh are affected by a given thermal cycle consisting of several to several points (steps) instead of the amount resulting from the density of the mesh and the size of the liquid metal pool (and hence also in some sense the dimensions of the heat source model). The cost incurred by the user in the event of its application results in the lower precision of the results obtained, caused by the specifics of thermal load (e.g., increases in stresses values at the end of the section into which the weld bead is divided (Figure 9b). Some differences also occur as a distribution of calculated quantities, although the maximum and minimum values and their distribution are usually similar to “transient” calculations results (Figure 8, Figure 9, Figure 10, Figure 13 and Figure 14).

In addition, in the case of resignation from calculations of the temperature fields and metallurgical phase distributions and the imposed heat cycle that was previously calibrated using a simple model calculated using the “transient” technique, it is observed a significant reduction of the calculation times. For the presented simulation examples, the calculation time reduction (without taking into account the calibration stage of the heat source model, which must also be carried out for the “transient” method at heat source calibration stage) is from a few to several times (Table 1, Table 2 and Table 3). In the case of very complex structures, where the use of the “transient” technique is impossible due to the aforementioned long calculation time, the use of the MBD DE or “shrinkage” technique supports the very fast prediction of the deformations of the weld structure. In this case, the abandonment of calculations of temperature field and metallurgical phase distribution and the use of shell elements instead of 3D solid elements, on the one hand, limits the result to the approximate deformation distribution, but on the other hand, it allows to quickly check different clamping variants and the order and direction welding individual beads. It can, therefore, be said that in a situation when, after proper calibration, the displacements distributions are similar, and the analysis mainly concerns structure deformations, the use of this computational technique brings measurable time benefits. This will be particularly evident when a large element or entire structure containing a large number of welds/beads is being analyzed. The mentioned repeatability of the welding parameters, allows for calibration on one model using the “transient” technique, and then transferring them to the structure and finally calculating the total deformation using the MBD or “shrinkage” technique. In such cases, the results of the analyses are significantly accelerated and this is often the only possible solution in terms of hardware and time limitations.

However, not only the mesh density or the complexity of the structure affects the calculation time. The type of material of the welded parts also affects the calculation time. It is because the whole simulation process is divided into time cards. Their amount depends on the density of the mesh, i.e., the number of steps that the moving heat source and cards related to the next cooling of the welded element to the set temperature must follow. Individual iterations in the calculation process of the selected time card must meet the assumed convergence criterion. Only then does the solver move to the next card. The number of iterations strictly depends on the non-linearity of the calculations. In turn, the non-linearity of the calculations is greater when the plastic deformations in the material are higher. Thus, the lower the yield strength of a material, the faster it gets into plastic deformations but also convergence problems. This is very well visible on the presented example of with laser welding of AISI 304 steel and Inconel 625 nickel superalloy pipes. The almost twice lower yield strength for AISI 304 steel makes it so that the calculation time is longer compared to the calculation time for Inconel 625 nickel superalloy. The differences in the calculation time for both materials in the case of the “transient” method are approximately 26% and 24% for the MBD technique (Table 3). The slight difference in the calculation times when using the MBD DE technique is confirmed by the fact that, in this case, they are not taken take into account with the distribution of metallurgical phases and changes in material properties, and hence stresses and strains values.

In summary, in a situation where currently the main challenge for the industry is to reduce the time needed for design work and research and development activities, the described modern calculations methods create possibilities to increase efficiency and reduce the waiting time for the final product. In addition, it is possible to detect positioning problems and incorrect design, placement or use of fasteners. If we add the possibility of detecting the risk of defects during assembly, there is a real advantage of using numerical analyses in the form of improving production efficiency as well as significant economic benefits.

## Figures and Tables

**Table 1:** Summary of the duration times of numerical analyses for tested calculation techniques of arc welding of a T-joint model with one fillet weld.

Transient	MBD (MBD DE) ^1^	Shrinkage
2h 44 min 2 s	12 min 10 s (119 s)	55 s

^1^ MBD DE—Macro-Bead Distortion Engineering for the “local–global” approach using the MBD calculation technique.

**Table 2:** Summary of the duration times of numerical analyses for tested calculation techniques of T-joint with three arc welded beads.

Transient	MBD	MBD DE ^1^
1h 8 min 2 s	20 min 26 s	11 min 28 s

^1^ MBD DE—Macro-Bead Distortion Engineering for the “local–global” approach using the MBD calculation technique.

**Table 3:** Summary of the duration times of numerical analyses for tested calculation techniques of laser-welded stainless steel and Inconel 625 nickel superalloy pipes.

	Transient	MBD	(MBD DE) ^1^
AISI 304	45 min 8 s	5 min 1s	50 s
Inconel 625	33 min 12 s	3 min 48 s	55 s

^1^ MBD DE—Macro-Bead Distortion Engineering for the “local–global” approach using the MBD calculation.
